# Ecological correlates of population genetics in *Linum suffruticosum*, an heterostylous polyploid and taxonomic complex endemic to the Western Mediterranean Basin

**DOI:** 10.1093/aobpla/plae027

**Published:** 2024-05-21

**Authors:** Maria Antònia Vanrell, Letícia R Novaes, Ana Afonso, Juan Arroyo, Violeta Simón-Porcar

**Affiliations:** Department of Plant Biology and Ecology, Faculty of Biology, University of Seville, 41012 Seville, Spain; Department of Plant Biology and Ecology, Faculty of Biology, University of Seville, 41012 Seville, Spain; Centre for Functional Ecology, Associate Laboratory TERRA, Department of Life Sciences, University of Coimbra, Coimbra, Portugal; Department of Plant Biology and Ecology, Faculty of Biology, University of Seville, 41012 Seville, Spain; Department of Plant Biology and Ecology, Faculty of Biology, University of Seville, 41012 Seville, Spain

**Keywords:** cytotype, heterostyly, *Linum*, microsatellites, polyploidy, reciprocity, Western Mediterranean

## Abstract

*Linum suffruticosum s.l.* is a taxonomic complex widespread in the Western Mediterranean basin. The complex is characterized by a high phenotypic and cytogenetic diversity, and by a unique three-dimensional heterostyly system that makes it an obligate outcrosser. We studied the patterns of genetic diversity and structure of populations throughout the entire distribution of *L. suffruticosum s.l.* with microsatellite markers. We analysed their relationships with various biological and ecological variables, including the morph ratio and sex organ reciprocity of populations measured with a novel multi-dimensional method. Populations consistently showed an approximate 1:1 morph ratio with high sex organ reciprocity and high genetic diversity. We found high genetic differentiation of populations, showing a pattern of isolation by distance. The Rif mountains in NW Africa were the most important genetic barrier. The taxonomic treatment within the group was not related to the genetic differentiation of populations, but to their environmental differentiation. Genetic diversity was unrelated to latitude, elevation, population size, niche suitability or breeding system. However, there was a clear influence of ploidy level on the genetic diversity of populations, and a seeming centre–periphery pattern in its distribution. Our results suggest that polyploidization events, high outcrossing rates, isolation by distance and important geographical barriers to gene flow have played major roles in the microevolutionary history of this species complex.

## Teaser

*Linum suffruticosum* s.l. is a polyploid and taxonomic complex in the Western Mediterranean that exhibits unique features such as three-dimensional heterostyly, requiring cross-pollination between floral morphs. Using microsatellite markers, we analysed the genetic diversity and structure across the entire distribution of the species, and assessed the influence of biological, geographical and environmental factors. We found balanced floral morph ratios linked to high genetic diversity across populations, influenced by ploidy level and a centre–periphery pattern. Genetic differentiation follows an isolation-by-distance pattern, with the Rif mountains serving as the most significant genetic barrier. The taxonomic treatment within the complex correlates with environmental rather than genetic differentiation. Polyploidy, cross-pollination and geographic isolation have profoundly shaped the evolution of this taxon.

## Introduction

The Western Mediterranean basin is a biodiversity hotspot with an exceptional habitat heterogeneity, an outstanding concentration of plant species, and a high level of endemism (Heywood 1995). Such biodiversity stands upon the suit of climatic, geologic and habitat fragmentation episodes that took place on the region from the late Tertiary to the Holocene (Médail and Quezel 1997), which also shaped the evolutionary history of endemic plant lineages in the area (e.g. Espindola *et al.* 2010; Fernández-Mazuecos and Vargas 2010; Santos-Gally *et al.* 2012; Martín-Hernanz *et al.* 2021). Several endemic plant lineages widely distributed in the Western Mediterranean basin constitute nowadays taxonomic complexes with high genetic, phenotypic and even ploidy variation across populations and species (e.g. Zozomová-Lihová *et al.* 2014; Tomasello and Oberprieler 2022). Unravelling the patterns of genetic diversity and differentiation in such complexes can help us understand the microevolutionary processes underlying the patterns of plant diversity in the region.

Polyploidy is considered a driver of speciation and rapid diversification (van de Peer *et al.* 2017, 2021) that, nevertheless, has shown inconsistent effects on Mediterranean plant diversity (Escudero *et al.* 2018; Marques *et al.* 2018). Indeed, the effects of polyploidization can be complex and have a variety of evolutionary consequences in plant lineages, also at the microevolutionary level. On the one hand, the formation of a new cytotype represents a potential reproductive barrier and, consequently, may imply a bottleneck leading to a reduction in genetic diversity. Isolated cytotypes could shift towards selfing preventing the exclusion of the minority cytotype (Levin 1975; van Drunen and Husband 2019) and, if viable, they may promote genetic differentiation (Chester *et al.* 2012; Levin 2013). On the other hand, if gene flow is maintained or restored after cytotype differentiation, potentially leading to new hybrids, populations may increase their genetic variability and success, and recombination may result in the homogenization of the populations, thereby maintaining diversity but not differentiation (Meimberg *et al.* 2009; Modliszewski and Willis 2012).

*Linum suffruticosum* L. *s.l.* (Linaceae) is a taxonomic and polyploid complex of perennial woody plants with distinctive heterostylous white flowers endemic to the western Mediterranean basin (Martínez-Labarga and Muñoz-Garmendia 2015). Populations are broadly distributed in shrublands on limestones and are well adapted to environmental conditions across wide latitudinal (ca. 27º-51ºN) and elevation (ca. 50–2500 m) ranges in the region (Afonso *et al.* 2023). This complex bears an enormous phenotypic variability and encompasses five major cytotypes (diploids, tetraploids, haxeaploids, octoploids and decaploids) with a basic chromosome number of 8 or 9 (Afonso *et al.* 2021). Such variation has been attributed to past events of chromosomal rearrangements, whole genome duplications, hybridization and introgression among populations (Martínez-Labarga and Muñoz-Garmendia 2015; Afonso *et al.* 2021). The different ploidy levels are distributed parapatrically, with several contact zones between cytotypes that might not be reproductively isolated, although mixed-ploidy populations are rarely found. This is expected to influence the genetic diversity and structure of these populations (Afonso *et al.* 2021). In the absence of clear diagnostic traits, the definition of taxonomic entities has been made through morpho-geographical divisions that do not correspond to cytotypes (Afonso *et al.* 2021). Historically, three taxa have been consensually accepted as distinct species within this complex, namely, *L. salsoloides* Lam., *L. appressum* Caball. and *L. suffruticosum* L. (Jahandiez and Maire 1932; Ockendon and Walters 1968; López González 1979). Nevertheless, the most recent taxonomic treatment identified more than 20 taxa (including species and subspecies) in the Iberian Peninsula alone (Martínez-Labarga and Muñoz-Garmendia 2015).

**Figure 1.**
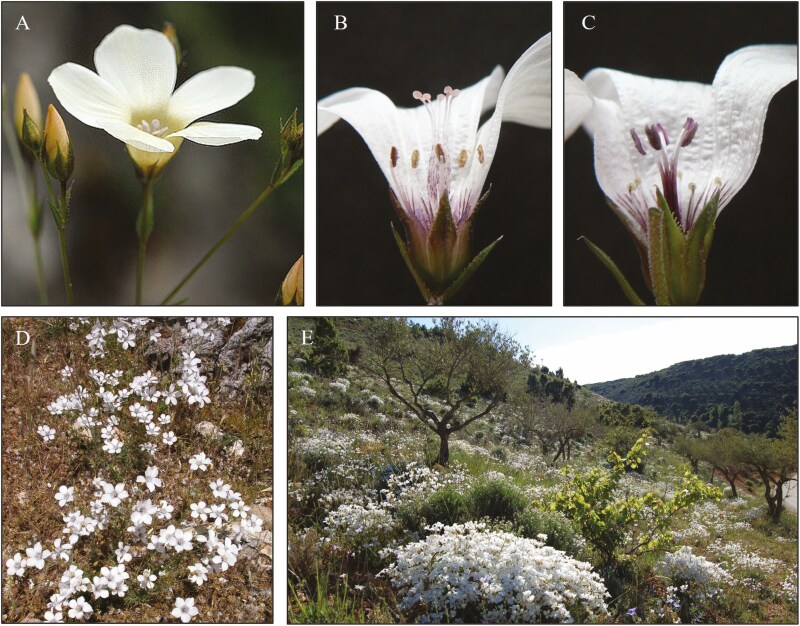
*Linum suffruticosum* flower (A), details for the long-styled (B) and short-styled (C) morphs, habit (D) and natural population (E).

*Linum suffruticosum s.l.* presents a unique floral polymorphism described as three-dimensional heterostyly (Armbruster *et al.* 2006). Heterostyly is defined by the reciprocal height of anthers and stigmas in two (distyly) or three (tristyly) morphs within a population, which improves pollen transfer between morphs through the segregation of pollen in frontal and distal parts of the pollinators’s body (Darwin 1863, 1877; Barrett *et al.* 2000). Beyond typical distyly, the two floral morphs of *L. suffruticosum s.l.* bear reciprocal stigmas and anthers not only in height but also in twisting and bending (Fig. 1). The result of this polymorphism is that, depending on the morph, the anthers and stigmas would contact the dorsal or ventral part of the pollinator, ensuring disassortative pollen transfer in a more effective way than typical distyly (Armbruster *et al.* 2006). In addition, three-dimensional heterostyly in *L. suffruticosum s.l.* seems to be associated with a heteromorphic self-incompatibility system (HetSI) that only allows fertilization between morphs (Nicholls 1985; Afonso 2022). Theoretically, HetSI allows complete disassortative mating and equal proportions of style morphs in heterostylous populations at equilibrium (isoplethy; Lloyd and Webb 1992). Conversely, biased morph ratios are often observed in heterostylous populations with relaxed HetSI, in whose case the sex organ reciprocity of morphs plays a key role in determining disassortative pollen transfer (Armbruster *et al.* 2009). Biased morph ratios appear also in populations subjected to stochastic factors (e.g. under unstable or changing pollination communities at higher elevations, latitudes or less suitable environments; Arroyo and Dafni 1995; Santos-Gally *et al.* 2013; or after habitat fragmentation with ensuing reduction of population sizes; Van Rossum *et al.* 2006). Unbalanced morph ratios reduce the abundance of compatible mates (i.e. the effective population size; van Rossum *et al.* 2006), and this may result in a loss of genetic diversity, with possible effects on the genetic differentiation of populations (Meeus *et al.* 2012). The occurrence of three-dimensional heterostyly across all *L. suffruticosum s.l.* cytotypes supports the missing effect of ploidy on this breeding system (Naiki 2012).

In this study, we analysed the patterns of genetic diversity and differentiation of *Linum suffruticosum s.l.* populations throughout the entire distribution range using nuclear microsatellite markers. We aimed to understand the evolutionary history underlying the intra-specific variation in this taxonomic and polyploid complex and to depict the possible role of heterostyly and environmental variation in it. We explored the relationships between the genetic pool and reproductive (morph ratio and sex organ reciprocity), biological (cytotype and taxonomic entity as a surrogate of morphological variation), demographic (population size), geographic (latitude and elevation) and environmental (climate and soil) traits of populations. To disentangle patterns of variation in reproductive traits, we also explored their association with biological, demographic, geographic and environmental traits. Based on theoretical expectations, we hypothesize that (i) the genetic pool of populations differs among ploidy levels and among taxonomic entities; (ii) reproductive traits do not differ among ploidy levels or taxonomic entities; (iii) lower levels of genetic diversity are associated to biased morph ratios, lower sex organ reciprocity, and/or reduced population sizes; (iv) given the broad environmental niche of the complex, elevation, latitude, and environmental suitability do not have a direct effect on the genetic diversity of populations, but they could have an indirect effect through their influence on reproductive traits, and (v) environmental differentiation of populations underlies the intra-specific variation of the complex. Overall, we aim to shed light on the microevolution of this group and, more broadly, on the genetic structuring patterns of Mediterranean polyploid species complexes and their interplay with heterostyly.

## Materials and Methods

### Population sampling

We sampled 32 *Linum suffruticosum s.l.* populations throughout the entire species’ range, from Northern Africa to the Maritime Alps across the eastern half of the Iberian Peninsula, during the 2018 to 2021 spring flowering seasons (see Supporting Information—Table S1). For the sake of simplicity, we will refer to *Linum suffruticosum s.l.* as *Linum suffruticosum* hereafter. For each population, we individually collected leaves from 12 individuals of known morph and stored them in silica gel. We estimated population size by counting plants across haphazardly chosen areas and extrapolating the number for the complete population. We estimated the morph ratio by visual determination of the floral morph of 60 randomly chosen flowers from different individuals (one flower per plant, *N* = 1427 flowers). In populations smaller than 60 individuals, all plants were checked. In 26 populations, we also measured the height and diameter of each sex organ whorl, and retrieved their *xy* coordinates to calculate the reciprocity index R2 of Sánchez *et al.* (2013) as modified by Simón-Porcar *et al.* (2024) for multidimensional data and implemented in the R package FlowerMate (Muñoz-Pajares and Simón-Porcar 2024). For 21 populations, we compiled available information on ploidy level (diploid to octoploid populations) and entities of putative taxonomic value (determined as *L. suffruticosum s.s.,* North African *L. suffruticosum,* or *L. appressum-salsoloides*) from Afonso *et al.* (2021). We determined the taxonomic entity of the remaining populations following the same criteria as in Afonso *et al.* (2021) (see Supporting Information—Table S1). We followed this taxonomic treatment because it allowed us to group populations in sufficient numbers for statistical analyses and because previous surveys on the species complex revealed serious difficulties in unambiguously assigning populations to taxa due to the existence of intermediate phenotypes (Afonso *et al.* 2021).

### Environmental characterization of populations

To characterize their ecology, we retrieved environmental information for each population from available databases and bibliography. We compiled 19 bioclimatic variables from the WorldClim database (https://www.worldclim.org), and 16 topographic and soil conditions variables at two different depths (15 and 30 cm) from the World Soil Information (https://www.isric.org), using the R package dismo (Hijmans *et al.*, 2017) (see Supporting Information—Table S2). We also retrieved data on the environmental niche suitability of each population from a recently published environmental niche model of *L. suffruticosum* (Afonso *et al*. 2023). This model included 324 records of natural populations of all cytotypes of this taxonomic complex and a subset of 14 of the above-mentioned environmental variables that were uncorrelated (Pearson’ *r* < 0.7; see details in Afonso *et al*. 2023).

### DNA isolation, SSR amplification and genotyping

Genomic DNA was extracted from the dried leaf samples (*N* = 382 individuals) in 2022 using the Invisorb® Spin Plant Mini Kit (Invitek Molecular GmbH, Germany). The concentration and quality of the extracted DNA were assessed with a Nanodrop DeNovix DS-11 Spectophotometer (DeNovix Inc., USA).

We used six specific *L. suffruticosum* SSR markers (Ls_1145191, Ls_337128, Ls_37372, Ls_395648, Ls_421659, Ls_9438; Olmedo *et al.* 2023) to genotype the samples. We performed simplex PCRs in 20 μL of master mix including: 1x MyTaq Red Reaction Buffer (Bioline), 0.40 μM dye-labelled M13 primer, 0.40 μM PIG-tailed reverse primer, 0.04 μM M13-tailed forward primer, 0.01% bovine serum albumin (BSA, Promega), 0.5 μL MyTaqTM Red DNA polymerase (Bioline), 50–70 ng gDNA and deionized water up to 20 μL. M13-tailed forward primers were labelled with either 6-FAM, VIC, NED, or PET fluorescent labels. The PCRs were performed in a Veriti™ 96-Well Thermal Cycler (Applied Biosystems™, USA) following the same touchdown procedure for all loci. This consisted of an initial denaturation for 2 min at 94 °C; followed by 10 cycles of 92 °C for 30 s, 30 s at 63 °C with an increment of −1 °C per cycle, and 30 s at 72 °C; followed by 20 cycles of 94 °C for 30 s, 30 s at 56 °C, and 30 s at 72 °C; and an extra extension of 5 min at 72 °C. Amplification products were analysed by MACROGEN (Madrid, Spain) in an automatic ABI 3730 capillary DNA sequencer with a GeneScan 500 LIZ internal size standard. The resulting electropherograms were analysed for allele binning and calling using PeakScanner™ Software V2.0 (Applied Biosystems™, USA).

### Genetic diversity

We used the retrieved genotypes to characterize the genetic diversity of *L. suffruticosum* populations. Given the polyploid nature of the studied complex, we used GenoDive V3.06 (Meirmans 2020) to calculate the standard metrics of genetic diversity per population and per locus: number of alleles (*N*_*a*_), effective number of alleles (*N*_*e*_), expected heterozygosity (*H*_*s*_) and total and corrected total heterozygosity (*H*_*t*_ and *H’*_*t*_), the last two only on a per locus basis. The *F*_*IS*_ statistic was used to test deviations from Hardy–Weinberg Equilibrium (HWE). GenoDive corrects for the unknown dosage of alleles in polyploids by a maximum likelihood method based on random mating within populations or by a method based on population allele frequencies, depending on the parameter. Since this correction is not applicable to the calculation of observed heterozygosity (*H*_*o*_) and inbreeding coefficient (*G*_*IS*_) statistics, we did not estimate these parameters. To assess the potential bias in genetic diversity indices caused by the small sample size per population (12 individuals) in our results, we performed a jackknife analysis by taking increasingly large subsamples of our data, from 5 to 12 individuals per population. Since the trend in the value of the different indices towards larger subsample sizes was relatively flat, we considered our sample size as representative (Supporting Information—Fig.S1).

### Genetic differentiation

We evaluated the genetic differentiation among populations, ploidy levels and taxonomic entities with analyses of molecular variance (AMOVA) run in GenoDive (Meirmans 2020). We tested the existence of isolation-by-distance with a Mantel test regressing population *F*_*ST*_ pairwise genetic distances (*Fst*/(1-*Fst*)) and the log-transformed geographic Euclidean distances between populations in the R package vegan 2.6-4 (Oksanen *et al.* 2022). *P*-values were estimated from 1000 permutations. Given the genetic differentiation found among ploidy levels, we carried out a second Mantel test to examine the correlation between *F*_*ST*_-based genetic distances and ploidy level-based distances (calculated as the pairwise difference between population ploidy levels; e.g. a value of six for a diploid—octoploid population pair).

### Genetic structure

A Bayesian clustering of individuals was performed using the STRUCTURE v.2.3.4 software (Pritchard *et al.* 2000), which has been shown to be the most suitable clustering method in mixed-ploidy systems (Stift *et al.* 2019). First, we estimated the population structure for the complete dataset using the admixture model and the locations as prior with a 2 × 10^4^ burn-in period and subsequent 1 × 10^5^ MCMC steps. We evaluated 1 ≤ *K* ≤ 10 potential genetic clusters (*K*) with 10 replicates for each *K* value. The optimal number of *K* was determined by the Evanno method (Evanno *et al.* 2005), checked with the aid of the Pophelper app (Francis 2017). As in this first clustering the optimal *K* equalled 2 (Supporting Information—Fig. S2a), and cluster 2 included only three populations, we repeated the analysis using the same parameters but including only the populations assigned to cluster 1. We generated graphical representations of the population genetic structure retrieved with the Pophelper app (Francis 2017). Finally, we performed a principal component analysis (PCA) based on a matrix of covariance of allele frequencies among all populations with GenoDive. We plotted the PCA results using the ggplot2 R package (Wickham 2016), grouping populations by STRUCTURE genetic clusters, ploidy level and taxonomic entity. To better visualize the relationships among population groups, we computed a Minimum Spanning Tree (MST) with the R package ape v.5.6 (Paradis and Schliep 2019) and added it to PCA plots.

### Patterns of genetic diversity

We tested whether the genetic diversity of populations (measured as *N*_*a*_, *N*_*e*_ and *H*_*s*_) differed among ploidy levels, taxonomic entities and genetic clusters retrieved by STRUCTURE with ANOVAs and Tukey HSD post-hoc tests ran in the stats R package (R Core Team 2021). In this and following analyses, we considered the three genetic clusters (cluster 1.1, cluster 1.2 and cluster 2) defined by the first clustering analysis (*K* = 2) and the clustering analysis within cluster 1 (*K* = 2). Three populations had unassigned taxonomic entities and one population had a mixture of diploid and triploid individuals. The analyses were repeated by assigning these populations to each putative group. Similar results were retrieved in all cases, and hence we report on the results for the analyses classifying such populations as *L. suffruticosum s.s.* and as diploid.

We also tested the association of genetic diversity with the reproductive (morph ratio and sex organ reciprocity), demographic (population size), and environmental (latitude, elevation, and niche suitability) traits of populations. Given the geographically structured nature of our data, we computed the Moran’s *I* statistic with ape v.5.6 to test the spatial autocorrelation of each variable. Only latitude showed significant spatial autocorrelation (Supporting Information—Table S3) but, to avoid spurious results, we used Dutilleul’s modified *t*-tests, as implemented in the software SAM v.4.0 (Rangel *et al.*, 2010), to analyse the correlations between each genetic diversity parameter and ecological variable. Given the multiple comparisons computed, we applied a Bonferroni correction to determine the significance threshold for *P*-values.

### Patterns of morph ratio and sex organ reciprocity

We tested whether the morph ratio of populations differed significantly from isoplethy (1:1 ratio) with proportion tests. We also tested the relationship between the morph ratio and sex organ reciprocity of populations, as well as the relationship of both variables with population size, latitude, elevation, and niche suitability using Dutilleul’s modified *t*-tests with the Bonferroni correction. Finally, we tested whether the morph ratio and sex organ reciprocity differed among ploidy levels, taxonomic entities, and genetic clusters using ANOVAs and Tukey HSD post hoc tests.

### Environmental differentiation of populations

We performed PCAs pooling all climatic, topographic and soil variables, and grouping populations in the PCA space by genetic cluster, ploidy level and taxonomic entity. We computed ANOVAs and Tukey HSD post hoc tests to estimate the differentiation among genetic clusters, ploidy levels and taxonomic entities in each individual climatic, topographic, and soil variable in our dataset, in the two first principal components of the environmental PCA, and in niche suitability. Given the multiple comparisons computed, we applied a Bonferroni correction to determine the significance threshold for *P*-values.

## Results

### Characterization of SSR markers

The six SSR markers analysed displayed 84 different alleles, with an average of 14 alleles per locus (range 10-30). Ls_337128 was the locus with the greatest genetic diversity, as besides having the highest number of alleles (*N*_*a*_=30), it had the highest effective number of alleles (*N*_*e*_ = 3.883) and the highest expected, total, and corrected total heterozygosity (*H*_*s*_ = 0.835, *H*_*t*_ = 0.924, *H’*_*t*_ = 0.927, respectively) (Supporting Information Table S4a).

### Genetic diversity

The average number of alleles in a population (*N*_*a*_) was 4.844, ranging from 2.167 (FLAX24) to 7.833 (FLAX78). Similarly, the average number of effective alleles in a population (*N*_*e*_) was 3.050, ranging from 1.709 (FLAX24) to 4.860 (FLAX78). The expected heterozygosity (*H*_*s*_) ranged from 0.381 (FLAX 61) to 0.744 (FLAX9), with an average of 0.584 (Supporting Information Table S4b). Based on the *F*_*IS*_ statistic, all populations significantly deviated from HWE (*P* < 0.001).

### Genetic differentiation

The genetic differentiation of populations followed a significant pattern of isolation-by-distance (Mantel test; *r = *0.319; *P* = 0.010). The analyses of molecular variance (AMOVA) revealed a significant differentiation among populations and among ploidy levels (Table 1a), but not among taxonomic entities (Table 1b). The majority (45.9%) of total genetic variation was found within populations, followed by variation among populations (42.2%), and among ploidy levels (11.9%). Nevertheless, there was no correlation between the genetic distance (based on *F*_*ST*_) and the ploidy differentiation of populations (Mantel test; *r = *-0.165; *P* = 0.970).

**Table 1.** Results of the analyses of molecular variance (AMOVA) grouping *L. suffruticosum* populations by ploidy levels (A) and by taxonomic entities (B).

Source of variation	%Var	*P* value
**(A)**		
Within populations	0.459	–
Among populations	0.422	<0.001*
Among ploidy levels	0.119	<0.001*
**(B)**		
Within populations	0.482	–
Among populations	0.521	<0.001*
Among taxonomic entities	0	0.579

### Genetic structure

**Figure 2.**
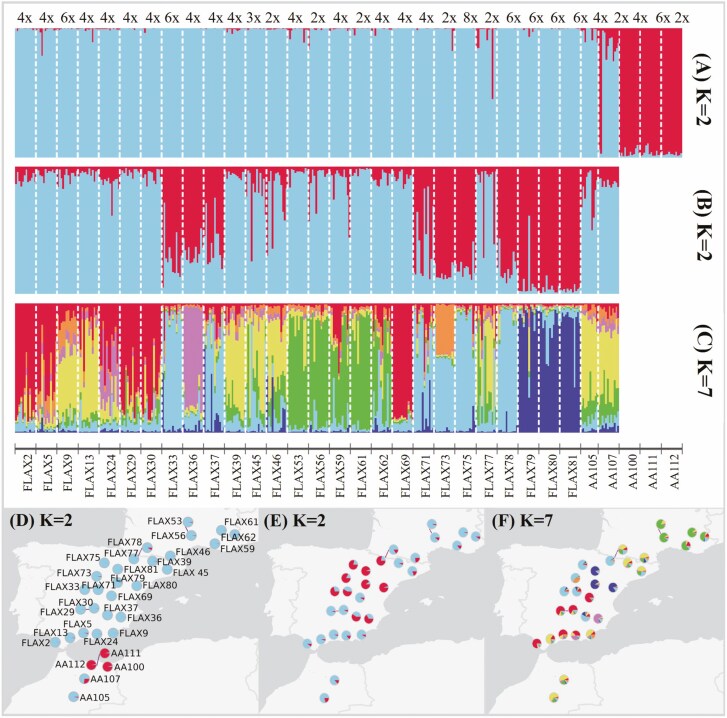
Genetic structure of *Linum suffruticosum* populations. Results of the Bayesian clustering of populations and geographic distribution of clusters, represented by different colours. Results for the most likely numbers of genetic clusters are shown: two genetic clusters when analysing the whole dataset (K = 2; A, D); and two (K = 2; B, E) and seven (K = 7; C, F) genetic clusters after excluding Riffan cluster from the analyses. The cytotype of each population is indicated above plot (A); population labelled as 3x had a mixture of diploid and triploid individuals.

**Figure 3.**
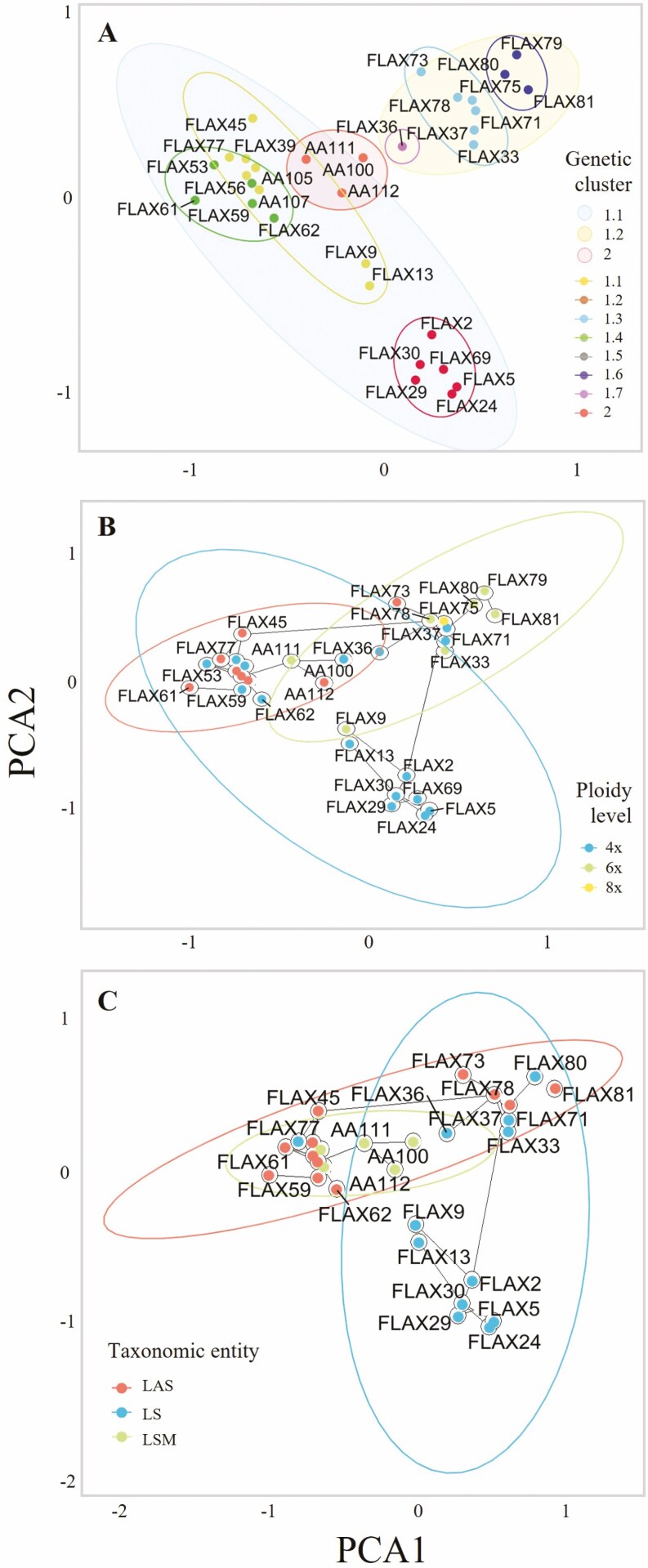
Results of the PCA analyses with populations grouped by STRUCTURE genetic cluster (A); ploidy level (B) and taxonomic entity (C). Plot (A) represents the three clusters resulting from K = 2 in both full datasets and excluding Riffan cluster in shadows; and the eight clusters resulting from K = 2 in the full dataset and K = 7 excluding Riffan cluster in empty circles. A Minimum Spanning Tree has been superimposed on plots (B) and (C). LS: *Linum suffruticosum s.s.*; LAS: *L. appressum*-*salsoloides*, LSM: North African *Linum suffruticosum*.

The STRUCTURE analyses found that Δ*K* was maximized at *K *= 2 (Supporting Information Fig. S2a), suggesting two distinct genetic clusters (Fig. 2a). Only three North African populations isolated by the eastern Rif mountains were assigned to cluster 2 (AA100, AA111 and AA112), and the other 29 populations were assigned to cluster 1 (Fig. 2b). When removing the three populations from cluster 2 from the analysis, the Δ*K* was maximized again at *K *= 2, and a second likelihood peak appeared at *K *= 7 (Supporting Information Fig. S2b), with the majority of clusters being geographically aggregated (Fig. 2b). The genetic PCA analysis showed moderately dense population groups, which paralleled the genetic clusters assigned by STRUCTURE (Fig. 3a). The ploidy level of populations presented only partial correspondence with the PCA clustering (Fig. 3b). PCA1 differentiated most diploid and hexaploid populations, although some populations of different cytotypes were aggregated in the PCA space. Furthermore, the MST tree connected populations of different cytotypes that were separated in the PCA space. These results support the multiple independent origins of polyploid populations across the range of *L. suffruticosum*. The three taxonomic entities did not show any clustering in the PCA space (Fig. 3c).

### Patterns of genetic diversity

**Figure 4.**
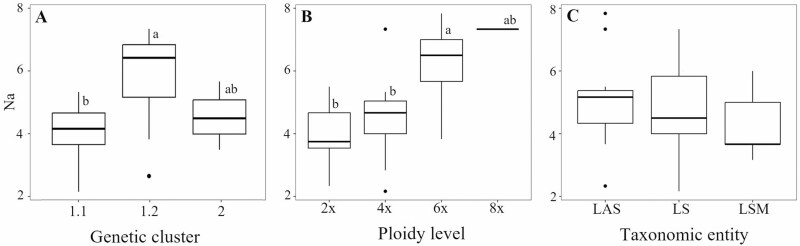
Genetic diversity (Na) of populations grouped by STRUCTURE genetic cluster (A), ploidy level (B) and taxonomic entity (C). Significant differences between groups according to the Tukey test are represented by different letters over each box in each plot. LS: *Linum suffruticosum s.s.*; LAS: *L. appressum-salsoloides*, LSM: North African *Linum suffruticosum*.

We found significant differences in genetic diversity among genetic clusters (*N*_*a*_ and *N*_*e*_) and among ploidy levels (*N*_*a*_, *N*_*e*_ and *H*_*s*_), but not among taxonomic entities (Fig. 4; Supporting Information Table S5). The clusters 1.1 and 1.2 showed the lowest and highest average values of genetic diversity per population, respectively (cluster 1.1: *N*_*a*_ = 4.219, *N*_*e*_ = 2.747; cluster 1.2: *N*_*a*_ = 6.016, *N*_*e*_ = 3.603), and the post hoc test showed a significant difference between them, but not with cluster 2 (Fig. 4a; Supporting Information Tables S6a and S7a). The genetic diversity increased with ploidy level for all analysed parameters (diploids: *N*_*a*_ = 3.979, *N*_*e*_ = 2.650, *H*_*s*_ = 0.530; octoploids: *N*_*a*_ = 7.333, *N*_*e*_ = 4.266, *H*_*s*_ = 0.689), and the Tukey HSD post hoc test showed significant differences between diploids and hexaploids and between tetraploids and hexaploids for at least one genetic diversity parameter (Fig. 4b; Supporting Information Tables S6b and S7b).

**Figure 5.**
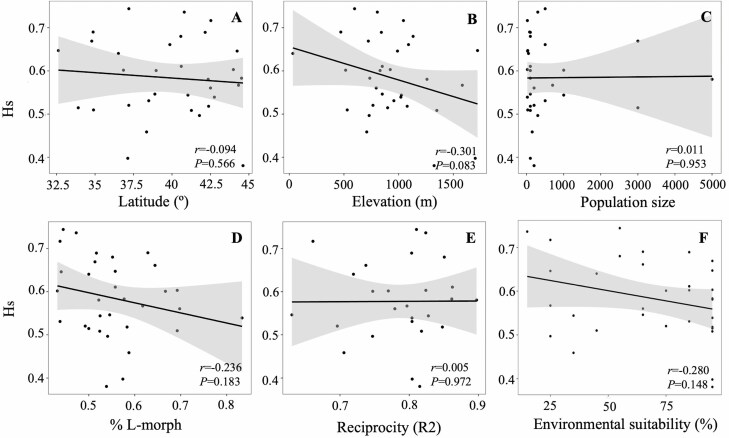
Scatter plots representing the linear regressions between the expected heterozygosity (H_S_) and latitude (A), elevation (B), population size (C), percentage of long-styled (L-) morph (D), reciprocity (R2) (E), and environmental suitability (F). *P*-values from Dutilleul’s modified *t*-tests are presented in each plot.

The genetic diversity of populations was not significantly correlated with any variable analysed (percentage of L-morph, sex organ reciprocity, population size, latitude, elevation, and niche suitability) (Pearson’s *r *< 0.424; *F* < 6.000; *P* > 0.021; Bonferroni-adjusted significant threshold for *P* = 0.008; Fig. 5; Supporting Information Table S8).

### Patterns of morph ratio and sex organ reciprocity

**Figure 6.**
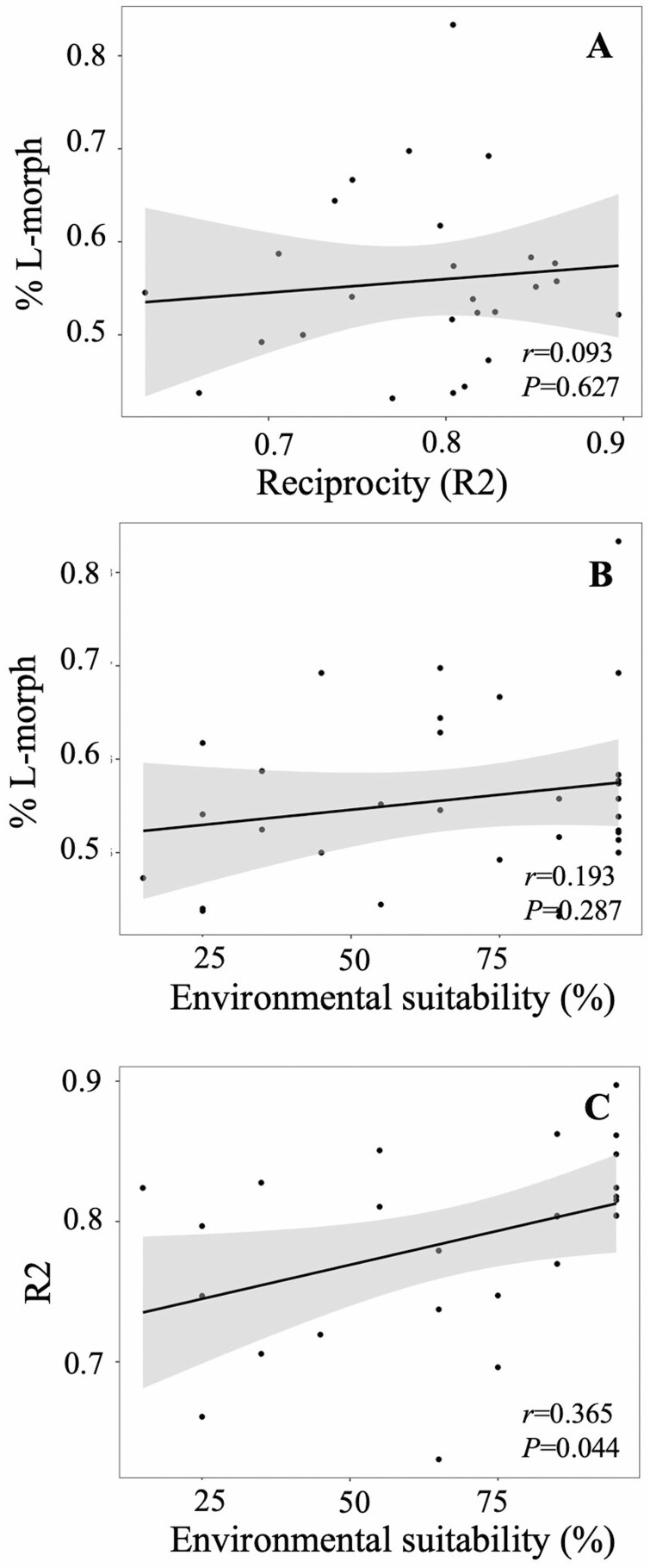
Scatter plots representing the linear regressions between the percentage of long-styled (L-) morph and reciprocity (R2) (A), between the percentage of long-styled (L-) morph and environmental suitability (B), and between reciprocity (R2) and environmental suitability (C). *P*-values from Dutilleul’s modified *t*-tests are presented in each plot.

Most populations showed a nearly 1:1 ratio of floral morphs (average proportion of L-morph = 0.557, range 0.432–0.833) and a high sex organ reciprocity (average *R*2 = 0.786, range 0.631–0.897). Only one population differed significantly from a 1:1 ratio (FLAX46; 83% L-morph; χ^2^* = *56.250; *P* < 0.001). The morph ratio and sex organ reciprocity of populations were uncorrelated (Pearson’s *r *= 0.093; *F* = 0.242; *P* = 0.627), nor they correlated with latitude, elevation, population size, or niche suitability (Pearson’s *r *< 0.365; *F* < 4.436; *P* > 0.044; Bonferroni-adjusted significant threshold for *P* = 0.010; Fig. 6; Supporting Information Table S9a). Populations of different ploidy levels, taxonomic entities and genetic clusters showed similar morph ratio and sex organ reciprocity (*F *< 3.033; *P* > 0.094; Supporting Information Table S9b).

### Environmental differentiation of populations

**Figure 7.**
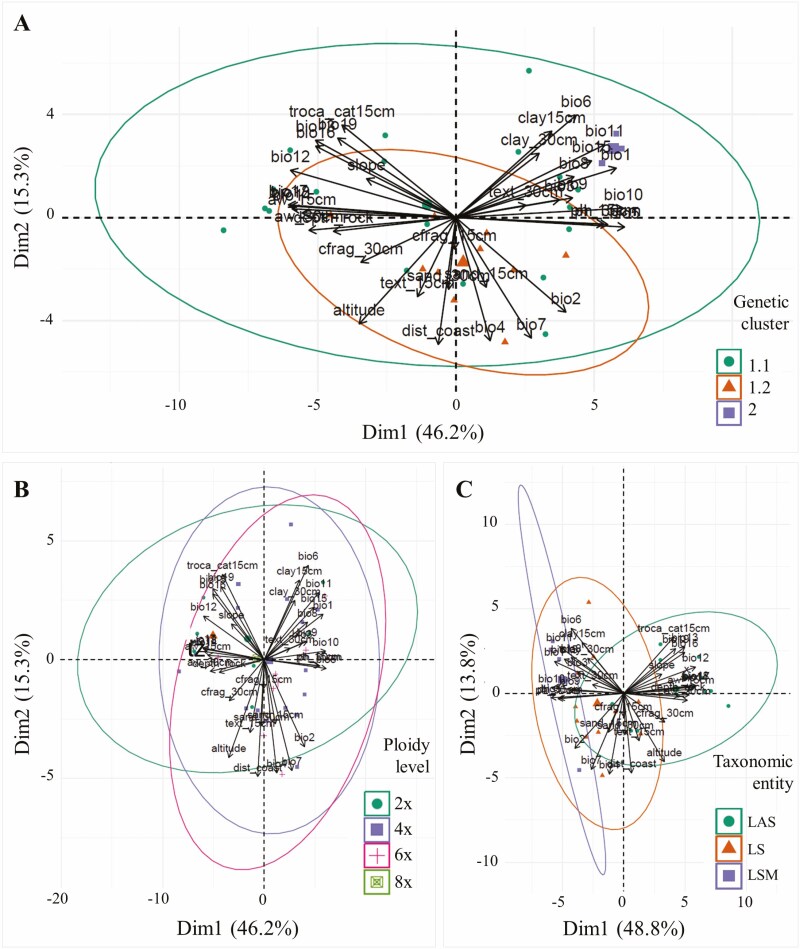
Environmental PCAs of populations of *Linum suffruticosum* grouped by STRUCTURE genetic cluster (A), ploidy level (B) and taxonomic entity (C). LS: *Linum suffruticosum s.s.*; LAS: *L. appressum-salsoloides*, LSM: North African *Linum suffruticosum*.

Populations of different genetic clusters showed incomplete clustering in the environmental PCA space. Clusters 1.2 and 2 were segregated from each other but not from Cluster 1.1 (Fig. 7a). After the Bonferroni correction (adjusted significant threshold for *P* = 0.001), only three environmental variables, and none PC, appeared significantly differentiated among genetic clusters (Supporting Information Table S10). Genetic clusters presented similar niche suitability (*F* = 2.196; *P* = 0.130).

Populations of different cytotypes were completely intermingled in the environmental PCA space (Fig. 7b), and all environmental individual variables, PC scores, and niche suitability were similar among them (*F* < 2.946; *P* > 0.050; Bonferroni-adjusted significant threshold for *P* = 0.001; Supporting Information Table S10).

Populations of different taxonomic entities showed seeming clustering along PC1 (Fig. 7c). Several environmental variables (27 of 36; 18 of 36 after Bonferroni correction) and PC1 differed significantly among taxonomic entities (*F* > 4.228; *P* < 0.025; Bonferroni-adjusted significant threshold for *P* = 0.001; Supporting Information Table S10). Taxonomic entities did not differ in niche suitability (*F* = 0.526; *P* = 0.597).

## Discussion

We explored the geographic, environmental and biological factors shaping the patterns of genetic diversity and structure in *Linum suffruticosum,* an heterostylous taxonomic and polyploid complex endemic to the western Mediterranean Basin. *Linum suffruticosum* populations show high levels of genetic diversity and significant differentiation across their wide distribution range, from North Africa to the Maritime Alps along the eastern half of the Iberian Peninsula (Fig. 1, Table 1 and Supporting Information Table S4). Former phylogenetic studies on the genus *Linum* (Maguilla *et al.* 2021) depicted a very recent (Pleistocene) origin and diversification of *L. suffruticosum*. Significantly, the species is absent in Balearic Islands, with abundant suitable habitats but already disconnected from the mainland since the Miocene. Hence, our results suggest a fast and dynamic range expansion, likely influenced by its highly effective outbreeding system and including intricate microevolutionary processes associated with numerous polyploidization events.

### *Genetic diversity of* L. suffruticosum *populations*

Across its range, *L. suffruticosum* presents abundant and frequently large populations, probably less affected by genetic drift (Frankham *et al.* 2002), which may contribute to the patterns of genetic diversity found. Nevertheless, the genetic diversity of study populations was independent of population size (Fig. 5c). This suggests that, despite the significant population differentiation and the isolation-by-distance pattern found, a sufficient gene flow (Loveless and Hamrick 1984), in combination with the complete outcrossing system (see below), might overcome the expected negative influence of small population size on genetic diversity.

The vast majority of *L. suffruticosum* populations, regardless of their cytotype, showed no significant deviations from the 1:1 ratio of floral morphs (isoplethy) and displayed a high sex organ reciprocity. These results suggest a complete disassortative mating across the range (Lau and Bosque 2003; Simón-Porcar *et al.* 2022), which would be assisted by the heteromorphic self-incompatibility system (Afonso 2022). These properties would also contribute to maintaining high levels of genetic diversity across the species range (Yuan *et al.* 2017). As a matter of fact, we did not find any association between morph ratio or sex organ reciprocity with any biological or ecological variable tested (Fig. 6 and Supporting Information Table S9). Stochastic events, selection, or relaxed HetSI commonly affect the morph ratios and, in turn, genetic diversity in other distylous species (Wedderburn and Richards 1990; van Rossum *et al.* 2006; van Rossum and Triest 2007; Meeus *et al.* 2012). Apparently, these processes do not play an important role in *L. suffruticosum*. Unfortunately, we lacked data to analyse sex organ reciprocity in North African populations, which will be required to confirm this result.

The genetic diversity of *L. suffruticosum* populations was not randomly distributed as our results support the existence of a centre-periphery pattern. Specifically, we found that the genetic cluster 1.2, located in the centre of the species distribution, showed significantly higher values of genetic diversity than cluster 1.1, distributed in the northern and southern ranges (Figs. 2 and 4). The centre–periphery pattern of diversity is relatively common in widely distributed species, in whose range limits populations may be subjected to less optimal abiotic and biotic conditions and/or lower levels of gene flow (Eckert *et al.* 2008; Pironon *et al.* 2017). We did not find an association between genetic diversity and the environmental suitability of populations, nor with their latitude or elevation (Fig. 5 and Supporting Information Table S8), also often related to sub-optimal and stochastic environments negatively impacting the demography and genetic diversity of populations in numerous species (Ellegren and Galtier 2016). These results suggest that environmental or geographical variables did not affect the genetic diversity of *L. suffruticosum* populations, which are well adapted to montane environments (Afonso *et al.* 2023). Hence, a lower gene flow in peripheral populations linked to the isolation-by-distance pattern may be the main driver of the centre–periphery pattern of genetic diversity in this species.

### *Genetic differentiation and structure of* L. suffruticosum populations

A significant pattern of isolation-by-distance influenced the genetic differentiation of *L. suffruticosum* populations, as shown by Mantel test and Bayesian clustering analyses. We found that study populations were structured in a mosaic of genetic clusters with a clear geographical component since populations belonging to each cluster were spatially aggregated (Fig. 2). The PCA highly supports this Bayesian structuring of populations (Fig. 3). We found that the eastern Rif mountains separate the two main genetic clusters of *L. suffruticosum*, as North African populations east of the Rif form a unique cluster and populations west of the Rif and Middle Atlas clustered with Iberian populations. Interestingly, this result matches the distribution of haplotypes retrieved from nuclear and plastid DNA sequences in previous studies in this taxon, supporting two independent lineages (Afonso *et al.* 2021). We highlight the singularity of the Riffan cluster, whose genetic differentiation and high genetic diversity, considering that it is composed of only three populations in our study, deserve further attention.

In contrast to other species in the region for which the Strait of Gibraltar is a major genetic barrier (e.g. Terrab *et al.* 2007; Arroyo *et al.* 2008; Escudero *et al.* 2008; Simón-Porcar *et al.* 2015), our results indicate that the western Rif has historically acted as the most significant genetic barrier for this species complex. Notably, this area is a centre of neoendemism within the Baetic-Rifan Super Hotspot ( Molina-Venegas *et al.* 2017). In fact, our results support the role played by the Strait of Gibraltar as migratory route for plants between both continents, paralleling the floristic homogeneity across the Super Hotspot (Valdés 1991; Molina-Venegas *et al.* 2015) and the genetic structure of other taxa distributed in the region (e.g. Brennan *et al.* 2014; Silva *et al.* 2015; Agudo *et al.* 2023). In contrast to older lineages, the putative age of *L. suffruticosum* (Pleistocene, more recent than the Messinian salinity crisis) testimonies the role of Pleistocene glaciations in connecting African and Iberian plates. The parallelisms arising from the amassed knowledge on the evolutionary history and genetic structure of several species in the region, and their ecological correlates, highlight its importance as a generator of plant diversity (Médail and Quezel 1997; Rodríguez-Sánchez *et al*. 2008; Lavergne *et al.* 2013; Simón-Porcar *et al.* 2018).

### *Polyploidy, taxonomy and genetic pool in* L. suffruticosum

The ploidy level had significant effects on the genetic pool of *L. suffruticosum* populations. On the one hand, there was a positive correlation between ploidy level with genetic diversity (Fig. 4); on the other hand, although we found an incomplete correspondence with the genetic structure of populations, there was a significant genetic differentiation among cytotypes (Table 1).

The formation of new cytotypes may entail bottlenecks and lower genetic diversity but, in contrast, we found that polyploidization significantly increased the genetic diversity of *L. suffruticosum* populations. This pattern is somehow superposed to the centre–periphery pattern exposed above, as most hexaploidy and octoploid populations belong to the geographically central cluster (Fig. 2). Nevertheless, the parapatric geographic distribution of *L. suffruticosum* cytotypes (Afonso *et al.* 2021) and the lack of correspondence with the genetic structuring of populations, as indicated by our Bayesian clustering, PCA and MST analyses (Figs. 2 and 3), suggest that they were formed repeatedly and independently from chromosomal rearrangements, whole genome duplications and hybridization events across the species distribution range. Such microevolutionary patterns should have allowed the incorporation of genetic diversity from different parental populations, which would explain the higher genetic diversity of polyploid populations (Soltis and Soltis 2000).

Despite polyploidization events taking place in all genetic clusters of *L. suffruticosum*, cytotypes presented significant genetic differentiation in AMOVA analyses (Table 1). This pattern, hinted by the scarcity of mixed polyploid populations, may partially parallel the isolation-by-distance pattern of genetic differentiation of populations given that the cytogenetic diversity of *L. suffruticosum* itself presents geographic segregation (Afonso *et al.* 2021), as commonly found in other polyploid complexes (Rice *et al.* 2019). Indeed, and despite our analyses of environmental variables with a lower number of populations did not detect it (Fig. 7 and Supporting Information Table S10), ploidy levels of *L. suffruticosum* occupy slightly different environmental niches and their hybridization at contact areas seems not common (Afonso *et al.* 2023), as observed in several other polyploid complexes (Comai 2005; Baniaga *et al.* 2020). This pattern suggests a balance between the processes that lead genetic differentiation and the maintenance of genetic diversity in *L. suffruticosum* cytotypes.

A variable number of taxonomic entities have been defined within *L. suffruticosum* based on morpho-geographical criteria in recurrent taxonomic treatments (Jahandiez and Maire 1932; Ockendon and Walters 1968; López González 1979; Martínez-Labarga and Muñoz-Garmendia 2015). Nevertheless, we found that even the taxa resulting from the simplest taxonomic treatment present similar levels of genetic diversity and are not genetically differentiated (Fig. 4 and Table 1). Although our sampling precluded such analyses, it seems reasonable to expect an even greater disassociation between genetic structure and taxa defined in the far most divisive treatment by Martínez-Labarga and Muñoz-Garmendia (2015). Despite that SSR are not the best genetic markers for taxonomic use, the incongruence found between the genetic differentiation of populations and their taxonomic classification should have a biological basis, calling for a reappraisal of the taxonomy of the group. It is noteworthy that taxonomic entities showed significant environmental differentiation in our analyses (Fig. 7 and Supporting Information Table S10), in contrast to genetic clusters. Common garden experiments may help unravel whether plasticity plays a relevant role in the phenotypic differentiation of taxonomic entities, which would advocate for a more conservative taxonomic treatment in this species complex.

### Concluding remarks

Reticulate microevolutionary processes, including multiple polyploidization events, an obligate outcrossing mating system, and distinctive geographical barriers to gene flow and migratory routes of the western Mediterranean geological history, have left a trace imprinted in the neutral genetic variation of the *L. suffruticosum* lineage. Given the recent origin of this lineage, our results support an active evolutionary history. Despite being unrelated to the phenotypic traits of taxonomic interest, the resulting intraspecific genetic variation should be critical in the adaptive processes underlying the occurrence of this species complex in different environments across its range, as well as in maintaining the evolutionary potential of the species in the face of current global change.

## Supporting Information

The following additional information is available in the online version of this article –

**Table S1.** Ecological and biological variables of *Linum suffruticosum* s.l. populations.

**Table S2.** Set of environmental variables compiled for the 32 study populations of *Linum suffruticosum*.

**Table S3.** Spatial autocorrelation of population variables.

**Table S4.** Genetic diversity parameters per locus (A) and population (B).

**Table S5.** Results of the ANOVAs on the differences in genetic diversity parameters among genetic clusters (A), ploidy levels (B) and taxonomic entities (C).

**Table S6.** Average genetic diversity population parameters for each genetic cluster (A), and ploidy level (B).

**Table S7.** Results of Tukey HSD post hoc testing pairwise differences in genetic diversity between genetic clusters (A) and ploidy levels (B).

**Table S8.** Relationship of genetic diversity with biological and ecological variables.

**Table S9.** Association of the reciprocity index and morph-ratio of populations with biological and ecological variables.

**Table S10.** Environmental differentiation of genetic clusters, ploidy levels and taxonomic entities.

**Figure S1.** Boxplots of genetic diversity indices averaged across populations, calculated from a jackknife analysis taking increasingly large subsamples of data.

**Figure S2.** Delta-K plots estimating the optimal number of genetic clusters following Evanno *et al.* (2005).

**Data S1.** Genotypes of 382 individuals of *Linum suffruticosum* s.l. retrieved with six microsatellite markers (A). Biological, geographic and environmental data for 32 populations of *Linum suffruticosum* s.l. (B).

plae027_suppl_Supplementary_Tables_S1-S10_Figures_S1-S2_Data_S1

## Data Availability

The data underlying this article are available in its online supplementary material.
