# Supplementary material for: Ecological correlates of population genetics in Linum suffruticosum, an heterostylous polyploid and taxonomic complex endemic to the Western Mediterranean Basin
Source: AoB Plants. 2024 May 21;16(4):plae027. doi: 10.1093/aobpla/plae027 (PMC11244263; doi:10.1093/aobpla/plae027)

Supporting material

**Table S1**. Ecological and biological variables of *Linum suffruticosum s.l.* populations. Pop: Population; Lat: latitude; Long: longitude; % L: Proportion of long-styled morph; Cluster: assigned cluster by STRUCTURE assuming two (K2; K=2 for the full dataset), three (K3; K=2 for the full dataset plus K=2 within Cluster 1) and eight (K8; K=2 for the full dataset plus K=7 within Cluster 1) clusters; R2: sexual reciprocity index. Taxa: *LS*: *Linum suffruticosum s.s.*; *LAS*: *L. appressum-salsoloides*, *LSM*: North African *Linum suffruticosum*; ID: Intermediate entity.

| Pop. Code | Lat. | Long. | Location | Ploidy | Taxa | % L | Elevation (m a.s.l) | Pop. size | Cluster (K2) | Cluster (K3) | Cluster (K8) | R2 |
| --- | --- | --- | --- | --- | --- | --- | --- | --- | --- | --- | --- | --- |
| FLAX2 | 36.516 | -6.138 | Cádiz, España | 4x | *LS* | 0.500 | 33 | 50 | 1 | 1.1 | 1.1 | 0.719 |
| FLAX5 | 37.194 | -4.122 | Granada, España | 4x | *LS* | 0.492 | 765 | 200 | 1 | 1.1 | 1.1 | 0.696 |
| FLAX9 | 37.198 | -1.999 | Almería, España | 6x | *LS* | 0.444 | 597 | 500 | 1 | 1.1 | 1.2 | 0.810 |
| FLAX13 | 36.846 | -4.805 | Málaga, España | 4x | *LS* | 0.432 | 515 | 1000 | 1 | 1.1 | 1.2 | 0.769 |
| FLAX24 | 37.127 | -3.451 | Granada, España | 4x | *LS* | 0.574 | 1700 | 100 | 1 | 1.1 | 1.1 | 0.804 |
| FLAX29 | 38.887 | -3.053 | Ciudad Real, España | 4x | *LS* | 0.545 | 850 | 100 | 1 | 1.1 | 1.1 | 0.631 |
| FLAX30 | 38.971 | -2.896 | Ciudad Real, España | 4x | *LS* | 0.667 | 830 | 100 | 1 | 1.1 | 1.1 | 0.747 |
| FLAX33 | 40.800 | -3.613 | Madrid, España | 6x | *LS* | 0.473 | 805 | 300 | 1 | 1.2 | 1.6 | 0.824 |
| FLAX36 | 38.349 | -1.993 | Albacete, España | 4x | *LS* | 0.587 | 708 | 150 | 1 | 1.2 | 1.3 | 0.706 |
| FLAX37 | 38.490 | -2.412 | Albacete, Spain | 4x | *LS* | 0.438 | 956 | 500 | 1 | 1.2 | 1.6 | 0.804 |
| FLAX39 | 42.381 | 0.791 | Lérida, Spain | 4x | *LAS* | 0.583 | 1078 | 100 | 1 | 1.1 | 1.2 | 0.848 |
| FLAX45 | 42.353 | 1.842 | Girona, Spain | 2x-3x | *LAS* | 0.522 | 1259 | 5000 | 1 | 1.1 | 1.2 | 0.897 |
| FLAX46 | 42.757 | 2.057 | Aude, France | 2x | *LAS* | 0.833 | 1020 | 20 | 1 | 1.1 | 1.2 | 0.804 |
| FLAX53 | 44.523 | 3.307 | Lozère, France | 4x | *LAS* | 0.577 | 736 | 100 | 1 | 1.1 | 1.4 | 0.861 |
| FLAX56 | 44.236 | 3.554 | Lozère, France | 2x | *LAS* | 0.440 | 989 | 25 | 1 | 1.1 | 1.4 | NA |
| FLAX59 | 43.992 | 5.242 | Vaucluse, France | 4x | *LAS* | 0.692 | 923 | 20 | 1 | 1.1 | 1.4 | 0.824 |
| FLAX61 | 44.619 | 6.174 | Hautes- France | 2x | *LAS* | 0.538 | 1324 | 200 | 1 | 1.1 | 1.4 | 0.815 |
| FLAX62 | 44.324 | 6.664 | Alpes-Provence, France | 4x | *LAS* | 0.617 | 1582 | 700 | 1 | 1.1 | 1.4 | 0.797 |
| FLAX69 | 39.863 | -2.135 | Cuenca, Spain | 4x | NA | 0.644 | 1090 | 500 | 1 | 1.1 | 1.1 | 0.737 |
| FLAX71 | 40.573 | -2.089 | Cuenca, Spain | 4x | *LAS* | 0.552 | 1132 | 100 | 1 | 1.2 | 1.6 | 0.851 |
| FLAX73 | 41.263 | -3.210 | Guadalajara, Spain | 2x | *LAS* | 0.524 | 1349 | 100 | 1 | 1.2 | 1.7 | 0.818 |
| FLAX75 | 42.157 | -2.109 | La Rioja, Spain | 8x | ID | 0.517 | 842 | 100 | 1 | 1.2 | 1.6 | 0.803 |
| FLAX77 | 42.504 | -0.349 | Huesca, Spain | 2x | *LS* | 0.698 | 795 | 200 | 1 | 1.1 | 1.2 | 0.779 |
| FLAX78 | 42.527 | -0.556 | Huesca, Spain | 6x | *LAS* | 0.438 | 1044 | 20 | 1 | 1.2 | 1.6 | 0.661 |
| FLAX79 | 41.038 | -1.709 | Zaragoza, Spain | 6x | NA | 0.525 | 1025 | 1000 | 1 | 1.2 | 1.5 | 0.828 |
| FLAX80 | 40.605 | -0.327 | Castellón, Spain | 6x | *LS* | NA | 850 | 100 | 1 | 1.2 | 1.5 | 0.862 |
| FLAX81 | 41.787 | -1.703 | Zaragoza, Spain | 6x | *LAS* | 0.541 | 730 | 300 | 1 | 1.2 | 1.5 | 0.747 |
| AA100 | 34.785 | -2.383 | Berka, Morocco | 4x | *LSM* | 0.514 | 723 | 3000 | 2 | 2 | 2 | NA |
| AA105 | 32.616 | -4.799 | Midelt, Morocco | 4x | *LSM* | NA | 1721 | 30 | 1 | 1.1 | 1.2 | NA |
| AA107 | 33.908 | -4.031 | Taza, Morocco | 2x | *LSM* | 0.5 | 899 | 3000 | 1 | 1.1 | 1.2 | NA |
| AA111 | 34.876 | -3.149 | Nador, Morocco | 6x | *LSM* | 0.629 | 471 | 80 | 2 | 2 | 2 | NA |
| AA112 | 34.901 | -3.547 | Driouch, Morocco | 2x | *LSM* | 0.692 | 530 | 30 | 2 | 2 | 2 | NA |

**Table S2.** Set of environmental variables compiled for the 32 study populations of *Linum suffruticosum*. The set includes 19 bioclimatic variables compiled from the WorldClim database and 16 topographic and soil conditions variables compiled from the World Soil Information.

| **Environmental variable** | **Code** |  |
| --- | --- | --- |
| Annual Mean Temperature | bio1 |  |
| Mean Diurnal Range (Mean of monthly (max temp - min temp)) | bio2 |  |
| Isothermality (BIO2/BIO7) (* 100) | bio3 |  |
| Temperature Seasonality (standard deviation *100) | bio4 |  |
| Max Temperature of Warmest Month | bio5 |  |
| Min Temperature of Coldest Month | bio6 |  |
| Temperature Annual Range (BIO5-BIO6) | bio7 |  |
| Mean Temperature of Wettest Quarter | bio8 |  |
| Mean Temperature of Driest Quarter | bio9 |  |
| Mean Temperature of Warmest Quarter | bio10 |  |
| Mean Temperature of Coldest Quarter | bio11 |  |
| Annual Precipitation | bio12 |  |
| Precipitation of Wettest Month | bio13 |  |
| Precipitation of Driest Month | bio14 |  |
| Precipitation Seasonality (Coefficient of Variation) | bio15 |  |
| Precipitation of Wettest Quarter | bio16 |  |
| Precipitation of Driest Quarter | bio17 |  |
| Precipitation of Warmest Quarter | bio18 |  |
| Precipitation of Coldest Quarter | bio19 |  |
| Elevation | ele |  |
| Derived available soil water capacity 15 cm | aw_15cm |  |
| Derived available soil water capacity 30 cm | aw_30cm |  |
| Cfrag_15cm | cfrag_15cm |  |
| Cfrag_30cm | cfrag_30cm |  |
| Clay content at 30 cm | clay_30cm |  |
| Clay content at 15 cm | clay15cm |  |
| Absolut depth to rock | depth_rock |  |
| Distance to the coast (metres) | dist_coast |  |
| pH at 15 cm | ph_15cm |  |
| pH at 30 cm | ph_30cm |  |
| Sand content 15 cm | sand_15cm |  |
| Sand content 30 cm | sand_30cm |  |
| Slope | slope |  |
| Texture at 15 cm | text_15cm |  |
| Texture at 30 cm | text_30cm |  |
| Cation exchange capacity 15 cm | troca_cat15cm |  |

**Table S3.** Spatial autocorrelation of population variables. N_a_: number of alleles per population; N_e_: effective number of alleles per population; H_s_: expected heterozygosity; % L-morph: proportion of long-styled morph individuals; R2: sexual reciprocity index.

| Parameter | Observed | Expected | s.d. | *P*-value |
| --- | --- | --- | --- | --- |
| N_a_ | 0.034 | -0.032 | 0.054 | 0.219 |
| N_e_ | -0.010 | -0.032 | 0.054 | 0.680 |
| H_S_ | -0.070 | -0.032 | 0.054 | 0.481 |
| Latitude | 0.461 | -0.032 | 0.054 | **< 0.001 ***** |
| Elevation | 0.032 | -0.032 | 0.053 | 0.220 |
| Niche suitability | -0.082 | -0.032 | 0.055 | 0.364 |
| Ploidy level | -0.002 | -0.032 | 0.054 | 0.580 |
| Pop size | -0.061 | -0.032 | 0.045 | 0.514 |
| % L-morph | -0.083 | -0.032 | 0.053 | 0.332 |
| R2 | 0.079 | -0.032 | 0.061 | 0.069 |
|  | | | | |

**Table S4.** Genetic diversity parameters per locus (A) and population (B). N_a_: number of alleles per locus; N_e_: effective number of alleles per locus; H_s_: expected Heterozygosity; Ht: total Heterozygosity; H't: corrected Total Heterozygosity.

| **(A)** Locus | N_a_ | N_e_ | H_s_ | H_t_ | H'_t_ |
| --- | --- | --- | --- | --- | --- |
| Ls_1145191 | 12 | 1.971 | 0.515 | 0.671 | 0.676 |
| Ls_337128 | 30 | 3.883 | 0.835 | 0.924 | 0.927 |
| Ls_37372 | 10 | 2.743 | 0.690 | 0.852 | 0.857 |
| Ls_395648 | 10 | 1.243 | 0.209 | 0.713 | 0.729 |
| Ls_421659 | 11 | 3.026 | 0.698 | 0.843 | 0.848 |
| Ls_9438 | 11 | 2.044 | 0.549 | 0.766 | 0.773 |
| **Overall** | **14 ± 7.188** | **2.485 ± 0.848** | **0.583 ± 0.197** | **0.795 ± 0.087** | **0.802 ± 0.084** |

|  |  |  |  |
| --- | --- | --- | --- |
| **(B)** Population | N_a_ | N_e_ | H_s_ |
| FLAX2 | 4.333 | 2.709 | 0.640 |
| FLAX5 | 4.000 | 2.494 | 0.520 |
| FLAX9 | 3.833 | 2.883 | 0.744 |
| FLAX13 | 4.167 | 2.413 | 0.602 |
| FLAX24 | 2.167 | 1.709 | 0.398 |
| FLAX29 | 5.000 | 2.926 | 0.546 |
| FLAX30 | 4.833 | 3.371 | 0.601 |
| FLAX33 | 7.167 | 4.533 | 0.736 |
| FLAX36 | 2.833 | 2.026 | 0.459 |
| FLAX37 | 5.167 | 3.282 | 0.531 |
| FLAX39 | 4.833 | 3.075 | 0.518 |
| FLAX45 | 5.500 | 2.841 | 0.581 |
| FLAX46 | 3.667 | 2.622 | 0.539 |
| FLAX53 | 4.500 | 3.050 | 0.583 |
| FLAX56 | 5.167 | 3.373 | 0.646 |
| FLAX59 | 5.333 | 3.590 | 0.603 |
| FLAX61 | 2.333 | 1.840 | 0.381 |
| FLAX62 | 5.167 | 3.332 | 0.567 |
| FLAX69 | 4.000 | 2.797 | 0.661 |
| FLAX71 | 7.333 | 4.200 | 0.680 |
| FLAX73 | 3.833 | 2.934 | 0.508 |
| FLAX75 | 7.333 | 4.266 | 0.689 |
| FLAX77 | 4.500 | 2.518 | 0.560 |
| FLAX78 | 7.833 | 4.860 | 0.717 |
| FLAX79 | 6.500 | 3.675 | 0.544 |
| FLAX80 | 6.833 | 3.448 | 0.611 |
| FLAX81 | 5.333 | 2.814 | 0.497 |
| AA100 | 5.000 | 2.988 | 0.669 |
| AA105 | 3.667 | 2.550 | 0.647 |
| AA107 | 3.167 | 2.117 | 0.515 |
| AA111 | 6.000 | 3.419 | 0.690 |
| AA112 | 3.667 | 2.955 | 0.510 |
| **Overall** | **4.844 ± 1.447** | **3.050 ± 0.731** | **0.584 ± 0.092** |

**Table S5**. Results of the ANOVAs on the differences in genetic diversity parameters among genetic clusters (A), ploidy levels (B) and taxonomic entities (C). N_a_: number of alleles; N_e_: effective number of alleles; H_s_: expected heterozygosity.

| 1. **Genetic clusters** | Df | Sum Sq | Mean Sq | *F-*value | *P*-value |
| --- | --- | --- | --- | --- | --- |
| N_a_ | 2 | 21.17 | 10.584 | 7.013 | **0.003 **** |
| N_e_ | 2 | 4.816 | 2.408 | 5.948 | **0.007 **** |
| H_s_ | 2 | 0.009 | 0.004 | 0.555 | 0.58 |
| **(B) Ploidy levels** |  |  |  |  |  |
| N_a_ | 3 | 26.99 | 8.997 | 6.639 | **0.001 **** |
| N_e_ | 3 | 5.705 | 1.902 | 4.907 | **0.007 **** |
| H_s_ | 3 | 0.064 | 0.021 | 3.132 | **0.041 *** |
| **(C) Taxonomic entities** |  |  |  |  |  |
| N_a_ | 2 | 2.12 | 1.058 | 0.481 | 0.623 |
| N_e_ | 2 | 0.693 | 0.346 | 0.607 | 0.552 |
| H_s_ | 2 | 0.005 | 0.002 | 0.308 | 0.738 |
|  |  |  |  |  |  |

**Table S6.** Average genetic diversity population parameters for each genetic cluster (A), and ploidy level (B). N_a_: number of alleles; N_e_: effective number of alleles; H_s_: expected heterozygosity.

| 1. Genetic cluster | N_a_ | N_e_ | H_s_ |
| --- | --- | --- | --- |
| 1.1 | 4.219 | 2.747 | 0.571 |
| 1.2 | 6.016 | 3.603 | 0.597 |
| 2 | 4.889 | 3.120 | 0.623 |
| 1. Ploidy level | N_a_ | N_e_ | H_s_ |
| 2x | 3.979 | 2.650 | 0.530 |
| 4x | 5.521 | 2.907 | 0.577 |
| 6x | 6.214 | 3.662 | 0.648 |
| 8x | 7.333 | 4.266 | 0.689 |

**Table S7.** Results of Tukey HSD post hoc testing pairwise differences in genetic diversity between genetic clusters (A) and ploidy levels (B). N_a_: number of alleles; N_e_: Effective number of alleles; H_s_: expected Heterozygosity.

| (A) Genetic clusters | | | | | | | | | |
| --- | --- | --- | --- | --- | --- | --- | --- | --- | --- |
| N_a_ | | Mean difference | | Lower 95% CI | | Upper 95% CI | | *P*-value | |
| 1.1 – 1.2 | | 1.797 | | 0.611 | | 2.982 | | **0.002 **** | |
| 1.1 – 2 | | 0.669 | | -1.215 | | 2.554 | | 0.658 | |
| 1.2 – 2 | | -1.127 | | -3.124 | | 0.869 | | 0.357 | |
| N_e_ | |  | |  | |  | |  | |
| 1.1 – 1.2 | | 0.855 | | 0.242 | | 1.469 | | **0.004 **** | |
| 1.1 – 2 | | 0.372 | | -0.603 | | 1.349 | | 0.617 | |
| 1.2 – 2 | | -0.483 | | -1.517 | | 0.551 | | 0.489 | |
| H_S_ | |  | |  | |  | |  | |
| 1.1 – 1.2 | | 0.026 | | -0.062 | | 0.114 | | 0.751 | |
| 1.1 – 2 | | 0.051 | | -0.089 | | 0.193 | | 0.641 | |
| 1.2 – 2 | | 0.025 | | -0.124 | | 0.175 | | 0.905 | |
|  | |  | |  | |  | |  | |
| (B) Ploidy levels | | | | | | | | |  |
| N_a_ | Mean difference | | Lower 95% CI | | Upper 95% CI | | *P*-value | |  |
| 4x – 2x | 0.541 | | -0.834 | | 1.917 | | 0.707 | |  |
| 6x – 2x | 2.235 | | 0.590 | | 3.880 | | **0.004 *** | |  |
| 8x – 2x | 3.354 | | -0.017 | | 6.725 | | 0.051 | |  |
| 6x – 4x | 1.693 | | 0.253 | | 3.133 | | **0.016 *** | |  |
| 8x – 4x | 2.812 | | -0.463 | | 6.088 | | 0.112 | |  |
| 8x – 6x | 1.119 | | -2.278 | | 4.516 | | 0.805 | |  |
| N_e_ |  | |  | |  | |  | |  |
| 4x – 2x | 0.256 | | -0.479 | | 0.992 | | 0.776 | |  |
| 6x – 2x | 1.011 | | 0.131 | | 1.891 | | **0.019 *** | |  |
| 8x – 2x | 1.616 | | -0.186 | | 3.419 | | 0.091 | |  |
| 6x – 4x | 0.754 | | -0.015 | | 1.524 | | 0.056 | |  |
| 8x – 4x | 1.359 | | -0.392 | | 3.111 | | 0.171 | |  |
| 8x – 6x | 0.604 | | -1.212 | | 2.421 | | 0.800 | |  |
| H_S_ |  | |  | |  | |  | |  |
| 4x – 2x | 0.046 | | -0.051 | | 0.144 | | 0.570 | |  |
| 6x – 2x | 0.118 | | 0.001 | | 0.235 | | 0.046 | |  |
| 8x – 2x | 0.159 | | -0.080 | | 0.399 | | 0.288 | |  |
| 6x – 4x | 0.071 | | -0.030 | | 0.174 | | 0.246 | |  |
| 8x – 4x | 0.112 | | -0.120 | | 0.345 | | 0.557 | |  |
| 8x – 6x | 0.041 | | -0.201 | | 0.282 | | 0.966 | |  |

**Table S8**. Relationship of genetic diversity with biological and ecological variables. Results of Dutilleul's modified t-tests for N_a_: number of alleles per population; N_e_: effective number of alleles per population; and H_S_: expected heterozygosity**.** Bonferroni-adjusted significant threshold for *P-*values = 0.008.

|  | Pearson’s *r* | | Corrected *F-value* |  | *P*-value |
| --- | --- | --- | --- | --- | --- |
| **% L-morph** |  |  | |  |  |
| N_a_ | -0.247 | 2.398 | |  | 0.130 |
| N_e_ | -0.189 | 1.356 | |  | 0.252 |
| H_S_ | -0.236 | 1.850 | |  | 0.183 |
| **Population size** |  |  | |  |  |
| N_a_ | -0.010 | 0.003 | |  | 0.956 |
| N_e_ | -0.189 | 1.120 | |  | 0.298 |
| H_S_ | 0.011 | 0.004 | |  | 0.953 |
| **Latitude** |  |  | |  |  |
| N_a_ | 0.287 | 1.497 | |  | 0.238 |
| N_e_ | 0.291 | 1.555 | |  | 0.230 |
| H_S_ | -0.094 | 0.336 | |  | 0.566 |
| **Elevation** |  |  | |  |  |
| N_a_ | -0.126 | 0.525 | |  | 0.474 |
| N_e_ | -0.095 | 0.289 | |  | 0.595 |
| H_S_ | -0.301 | 3.194 | |  | 0.083 |
| **Reciprocity (R2)** |  |  | |  |  |
| N_a_ | 0.128 | 0.456 | |  | 0.505 |
| N_e_ | 0.069 | 0.136 | |  | 0.715 |
| H_S_ | 0.005 | 0.001 | |  | 0.972 |
| **Niche suitability** |  |  | |  |  |
| N_a_ | -0.365 | 4.338 | |  | 0.045 |
| N_e_ | -0.424 | 6.000 | |  | 0.021 |
| H_S_ | -0.280 | 2.221 | |  | 0.148 |
|  |  |  | |  |  |

**Table S9.** Association of the reciprocity index and morph-ratio of populations with biological and ecological variables. (A) Results of the linear regressions testing the association of the reciprocity index (R2) and morph-ratio (%L) with ecological variables. Bonferroni-adjusted significant threshold for *P-*values = 0.01. (B) Results of the ANOVAs on the differences in the reciprocity index (R2) and morph-ratio (%L) among ploidy levels, taxonomic entities, and genetic clusters.

| **(A)** | Pearson’s *r* | Corrected *F-value* | *P*-value |
| --- | --- | --- | --- |
| **Reciprocity (R2)** |  |  |  |
| % L-morph | 0.093 | 0.242 | 0.627 |
| Latitude | 0.276 | 1.173 | 0.297 |
| Elevation | 0.276 | 1.843 | 0.188 |
| Population size | 0.288 | 2.790 | 0.105 |
| Niche suitability | 0.365 | 4.436 | 0.044 |
| **% L-morph** |  |  |  |
| Latitude | 0.146 | 0.628 | 0.434 |
| Elevation | 0.086 | 0.217 | 0.645 |
| Population size | -0.217 | 1.547 | 0.223 |
| Niche suitability | 0.193 | 1.175 | 0.287 |

| **(B)** | Df | Sum Sq | Mean Sq | *F*-value | *P*-value |
| --- | --- | --- | --- | --- | --- |
| **Reciprocity (R2)** |  |  |  |  |  |
| Ploidy level | 3 | 0.010 | 0.003 | 0.788 | 0.513 |
| Taxonomic entity | 1 | 0.011 | 0.011 | 3.033 | 0.094 |
| Genetic cluster | 1 | 0.000 | 0.000 | 0.060 | 0.808 |
| **% L-morph** |  |  |  |  |  |
| Ploidy level | 3 | 0.024 | 0.008 | 0.981 | 0.416 |
| Taxonomic entity | 2 | 0.009 | 0.004 | 0.543 | 0.587 |
| Genetic cluster | 2 | 0.030 | 0.015 | 1.929 | 0.164 |

**Table S10.** Environmental differentiation of genetic clusters, ploidy levels and taxonomic entities. We provide average ± s.d. values of environmental variables and the results of ANOVAs analysing differences among population groups. Different letter superscripts indicate significant differentiation among population groups based on Tukey HSD post hoc tests. We present results for each population grouping and each of 36 climate and soil variables separately, and for the first and second axes of the environmental PCA, and environmental suitability values.

|  |  | Genetic cluster | | | |  | Ploidy level | | | | |  | Taxonomic entity | | | |
| --- | --- | --- | --- | --- | --- | --- | --- | --- | --- | --- | --- | --- | --- | --- | --- | --- |
| Environmental variable |  | 1_1 | 1_2 | 2 | *F*-value |  | 2x | 4x | 6x | 8x | *F*-value |  | LSM | LS | LAS | *F*-value |
|  |  |  |  |  |  |  |  |  |  |  |  |  |  |  |  |  |
| Elevation |  | 980.5 ± 97.9 | 989.6 ± 61.7 | 531.3 ± 88.8 | 2.197 |  | 1026.7 ± 108.7 | 954.6 ± 112.8 | 818.1 ± 90.6 | 906 | 0.388 |  | 842.2 ± 235.3 | 847.8 ± 90.9 | 1099.4 ± 82.8 | 1.856 |
|  |  |  |  |  |  |  |  |  |  |  |  |  |  |  |  |  |
| Derived available soil water capacity 15 cm |  | 13.7 ± 0.5^b^ | 13 ±  0.2^ab^ | 10.6 ± 0.3^a^ | 3.843 * |  | 13.8 ±  0.9 | 13.0 ± 0.5 | 12.8 ± 0.4 | 13 | 0.338 |  | 11.2 ± 0.3^a^ | 12.4 ± 0.3^a^ | 15 ± 0.4^b^ | **17.042 ***** |
|  |  |  |  |  |  |  |  |  |  |  |  |  |  |  |  |  |
| Derived available soil water capacity 30 cm |  | 11.5 ± 0.3^b^ | 11.2 ± 0.3^ab^ | 9.3 ±  0.3^a^ | 4.019 * |  | 11.3 ±  0.5 | 11.3 ± 0.3 | 11 ±  0.5 | 11 | 0.098 |  | 10 ±  0.5^a^ | 10.6 ± 0.2^a^ | 12.5 ± 0.2^b^ | **14.712 ***** |
|  |  |  |  |  |  |  |  |  |  |  |  |  |  |  |  |  |
| Annual Mean Temperature |  | 11.7 ±  0.8 | 11.2 ±  0.5 | 16 ±  0.5 | 3.265 |  | 10.6 ±  1.1 | 12.4 ± 0.8 | 12.7 ± 1.0 | 10 | 0.866 |  | 15.2 ± 0.6^b^ | 13.2 ± 0.6^b^ | 9.1 ± 0.5^a^ | **19.789 ***** |
|  |  |  |  |  |  |  |  |  |  |  |  |  |  |  |  |  |
| Mean Temperature of Warmest Quarter |  | 19.7 ±  0.8 | 19.8 ±  0.7 | 23 ±  0.5 | 1.304 |  | 18.2 ±  1.1 | 20.8 ± 0.8 | 20.8 ± 0.9 | 18 | 1.391 |  | 22.8 ± 0.3^b^ | 21.8 ± 0.6^b^ | 16.7 ± 0.5^a^ | **26.163 ***** |
|  |  |  |  |  |  |  |  |  |  |  |  |  |  |  |  |  |
| Mean Temperature of Coldest Quarter |  | 4.5 ±  0.7^a^ | 4.2 ±  0.4^a^ | 10 ±  0.5^b^ | 5.682 ** |  | 3.7 ±  1.1 | 5.3 ±  0.8 | 5.7 ±  1.0 | 4 | 0.615 |  | 8.2 ± 1.2^b^ | 5.9 ± 0.6^b^ | 2.4 ± 0.4^a^ | **13.359 ***** |
|  |  |  |  |  |  |  |  |  |  |  |  |  |  |  |  |  |
| Annual Precipitation |  | 689.6 ± 63.6^b^ | 498.2 ± 35.1^ab^ | 325.6 ± 20.3^a^ | 4.654 * |  | 756.6 ± 106.1 | 584.4 ± 59.2 | 448 ± 57.8 | 523 | 2.122 |  | 313.4 ± 31.1^a^ | 496.4 ± 26.8^a^ | 837.4 ± 65.3^b^ | **24.615 ***** |
|  |  |  |  |  |  |  |  |  |  |  |  |  |  |  |  |  |
| Precipitation of Wettest Month |  | 87.4 ±  7.4 | 62.2 ±  3.3 | 50.3 ± 2.6 | 4.595 |  | 91.2 ± 12.6 | 76.5 ± 7.2 | 59 ±  5.1 | 68 | 1.634 |  | 45.6 ± 4.8^a^ | 67.4 ± 4.9^a^ | 99.6 ± 7.9^b^ | **12.834 ***** |
|  |  |  |  |  |  |  |  |  |  |  |  |  |  |  |  |  |
| Precipitation of Driest Month |  | 25.4 ±  5.2 | 20.8 ±  2.8 | 2.3 ±  0.3 | 2.005 |  | 34.7 ±  8.2 | 16.3 ± 4.4 | 18.7 ± 4.7 | 28 | 1.883 |  | 3.4 ± 0.6^a^ | 12.4 ± 2.6^a^ | 41.1 ± 4.3^b^ | **27.266 ***** |
|  |  |  |  |  |  |  |  |  |  |  |  |  |  |  |  |  |
| Precipitation Seasonality (CV) |  | 36.6 ± 3.9^a^ | 32.4 ± 1.8^a^ | 59.3 ± 2.7^b^ | 4.244 * |  | 31.2 ±  5.9 | 41.7 ± 3.9 | 36.1 ± 4.9 | 28 | 0.965 |  | 52.4 ± 5.1^b^ | 42.9 ± 3.7^b^ | 24.4 ± 1.9^a^ | **13.366 ***** |
|  |  |  |  |  |  |  |  |  |  |  |  |  |  |  |  |  |
| Precipitation of Wettest Quarter |  | 234.1 ± 19.1^b^ | 167.4 ± 9.1^a^ | 128.3 ± 8.8^a^ | 5.195 * |  | 240.3 ± 30.6 | 208.5 ± 19.3 | 153.1 ± 14.2 | 176 | 1.837 |  | 118.6 ± 12.7^a^ | 184.1 ± 14.1^a^ | 262.6 ± 19.8^b^ | **12.452 ***** |
|  |  |  |  |  |  |  |  |  |  |  |  |  |  |  |  |  |
| Precipitation of Driest Quarter |  | 102.4 ± 17.8 | 77.4 ±  9 | 15.3 ± 0.6 | 2.605 |  | 130.2 ± 28.5 | 71.8 ± 15.8 | 69.2 ± 15.9 | 91 | 1.676 |  | 20.6 ± 3.2^a^ | 53.8 ± 8.2^a^ | 154.6 ± 14.8^b^ | **31.1 ***** |
|  |  |  |  |  |  |  |  |  |  |  |  |  |  |  |  |  |
| Precipitation of Warmest Quarter |  | 107.0 ± 18.4 | 82.9 ±  9.5 | 23 ±  1.5 | 2.254 |  | 135.6 ± 29.7 | 75.8 ± 16.1 | 77.5 ± 16.6 | 91 | 1.579 |  | 25.2 ± 1.6^a^ | 59.5 ± 8.9^a^ | 159.4 ± 16.1^b^ | **26.037 ***** |
|  |  |  |  |  |  |  |  |  |  |  |  |  |  |  |  |  |
| Precipitation of Coldest Quarter |  | 200.4 ± 15.8^b^ | 129.8 ± 11.4^a^ | 123 ± 11.5^ab^ | 5.940 ** |  | 203.8 ± 22.3 | 181.4 ± 16.8 | 115.7 ± 15.0 | 132 | 2.942 |  | 109.2 ± 16.8^a^ | 158.8 ± 15.5^ab^ | 212.2 ± 17.5^b^ | 6.22 ** |
|  |  |  |  |  |  |  |  |  |  |  |  |  |  |  |  |  |
| Mean Diurnal Range (Mean monthly (max temp - min temp)) |  | 10.5 ±  0.3 | 11.7 ±  0.3 | 11.3 ± 0.3 | 2.524 |  | 10.2 ±  0.2 | 11.1 ± 0.3 | 11.4 ± 0.5 | 11 | 1.147 |  | 11.4 ± 0.2^ab^ | 11.6 ± 0.3^b^ | 10 ±  0.2^a^ | 7.325 ** |
|  |  |  |  |  |  |  |  |  |  |  |  |  |  |  |  |  |
| Isothermality (BIO2/BIO7) (* 100) |  | 38.2 ± 0.3^a^ | 40 ±  0.6^b^ | 43.3 ± 0.3^c^ | **13.837 ***** |  | 38.6 ±  0.7 | 38.9 ± 0.5 | 40.4 ± 0.9 | 41 | 1.177 |  | 41.4 ± 1.2^b^ | 39.3 ± 0.4^ab^ | 38.2 ± 0.5^a^ | 4.228 * |
|  |  |  |  |  |  |  |  |  |  |  |  |  |  |  |  |  |
| Temperature Seasonality (s.d. *100) |  | 626.3 ± 15.6^b^ | 638.3 ± 15.8^b^ | 526.6 ± 3.6^a^ | 4.116 * |  | 595.7 ± 14.8 | 639.7 ± 19.8 | 611.1 ± 20.0 | 584 | 0.962 |  | 590.8 ± 41.4 | 650.4 ± 18.3 | 596.1 ± 7.2 | 3.232 |
|  |  |  |  |  |  |  |  |  |  |  |  |  |  |  |  |  |
| Max Temperature of Warmest Month |  | 25.5 ±  0.9 | 25.9 ±  0.8 | 29 ±  0.5 | 1.222 |  | 23.8 ±  1.2 | 26.7 ± 0.9 | 26.8 ± 1.0 | 24 | 1.452 |  | 28.8 ± 0.3^b^ | 28 ± 0.6^b^ | 22.2 ± 0.6^a^ | **28.766 ***** |
|  |  |  |  |  |  |  |  |  |  |  |  |  |  |  |  |  |
| Min Temperature of Coldest Month |  | -2.2 ± 0.8^a^ | -3.3 ±  0.3^a^ | 3.3 ±  0.6^b^ | 6.134 ** |  | -2.7 ±  1.1 | -1.8 ± 0.9 | -1.4 ± 1.1 | -4 | 0.302 |  | 1.2 ± 1.4^b^ | -1.5 ± 0.9^ab^ | -4.1 ± 0.4^a^ | 6.391 ** |
|  |  |  |  |  |  |  |  |  |  |  |  |  |  |  |  |  |
| Temperature Annual Range (BIO5-BIO6) |  | 27.6 ±  0.7 | 29.3 ±  0.7 | 26 ±  0.5 | 1.727 |  | 26.6 ±  0.5 | 28.6 ± 0.9 | 28.4 ± 0.8 | 27 | 0.831 |  | 27.8 ± 1.2^ab^ | 29.5 ± 0.8^b^ | 26.2 ± 0.4^a^ | 4.828 * |
|  |  |  |  |  |  |  |  |  |  |  |  |  |  |  |  |  |
| Mean Temperature of Wettest Quarter |  | 8.7 ±  0.6 | 9.8 ±  0.9 | 12.6 ± 0.3 | 2.576 |  | 8.3 ±  1.1 | 9 ±  0.7 | 11.2 ± 0.9 | 12 | 1.689 |  | 12.2 ± 0.3^b^ | 9.9 ± 0.6^ab^ | 7.6 ± 0.9^a^ | 5.808 ** |
|  |  |  |  |  |  |  |  |  |  |  |  |  |  |  |  |  |
| Mean Temperature of Driest Quarter |  | 18.3 ±  1.5 | 16.8 ±  2.0 | 22.6 ± 0.3 | 0.983 |  | 16.5 ±  2.3 | 19.9 ± 1.3 | 16.4 ± 3.0 | 18 | 0.751 |  | 22.6 ± 0.2^b^ | 19.8 ± 1.6^ab^ | 14.5 ± 1.7^a^ | 4.548 * |
|  |  |  |  |  |  |  |  |  |  |  |  |  |  |  |  |  |
| Cfrag_15cm |  | 18.7 ±  0.9 | 18.4 ±  0.6 | 19.3 ± 0.8 | 0.087 |  | 18.1 ±  1.4 | 19.3 ± 0.8 | 17.8 ± 0.6 | 19 | 0.403 |  | 19.6 ± 0.5 | 18.9 ± 0.7 | 18.1 ± 1.2 | 0.507 |
|  |  |  |  |  |  |  |  |  |  |  |  |  |  |  |  |  |
| Cfrag_30cm |  | 17.6 ± 1.4^b^ | 18.5 ± 0.7^b^ | 4 ±  0^a^ | **14.245 ***** |  | 14.3 ±  2.6 | 17.7 ± 1.5 | 16.1 ± 2.1 | 20 | 0.589 |  | 4.6 ± 0.6^a^ | 18.9 ± 0.8^b^ | 18.7 ± 1.3^b^ | **47.961 ***** |
|  |  |  |  |  |  |  |  |  |  |  |  |  |  |  |  |  |
| Clay content at 30 cm |  | 27.8 ±  1.1 | 26.2 ±  1.2 | 27 ±  0 | 0.419 |  | 26.5 ±  0.8 | 28 ±  1.3 | 25.8 ± 1.5 | 31 | 0.657 |  | 26.8 ± 0.4 | 28.6 ± 1.2 | 25.7 ± 1.3 | 1.484 |
|  |  |  |  |  |  |  |  |  |  |  |  |  |  |  |  |  |
| Clay content at 15 cm |  | 25.1 ± 0.8^a^ | 22.9 ± 0.9^a^ | 31 ±  0.5^b^ | 5.951 ** |  | 25.7 ±  1.5 | 25.1 ± 0.9 | 23.4 ± 1.6 | 26 | 0.466 |  | 30.4 ± 1.2^b^ | 24.1 ± 0.9^a^ | 23.7 ± 0.9^a^ | 7.238 ** |
|  |  |  |  |  |  |  |  |  |  |  |  |  |  |  |  |  |
| Absolut depth to rock |  | 1289.4 ± 226.6 | 1329.9 ± 203.6 | 108.3 ± 88.6 | 2.643 |  | 1402.5 ± 302.9 | 1064.3 ± 249.0 | 1175 ± 323.5 | 1649 | 0.316 |  | 694.6 ± 478.8^a^ | 818.6 ± 200.2^a^ | 1864.2 ± 162.5^b^ | 7.691 ** |
|  |  |  |  |  |  |  |  |  |  |  |  |  |  |  |  |  |
| Distance to the coast (metres) |  | 138748 ± 19675^ab^ | 213682 ± 27368^b^ | 42746.7 ± 1427.1^a^ | 5.598 ** |  | 154785.0 ± 27819.9 | 145192 ± 23422 | 167246 ± 48619 | 169203 | 0.091 |  | 118411 ± 50204 | 152371 ± 27990 | 168639 ± 20058 | 0.483 |
|  |  |  |  |  |  |  |  |  |  |  |  |  |  |  |  |  |
| pH at 15 cm |  | 69.1 ± 1.3^a^ | 70.7 ±  2^ab^ | 79 ±  1^b^ | 3.792 * |  | 68.3 ±  2.6 | 71.3 ± 1.2 | 72 ±  2.9 | 66 | 0.682 |  | 78.8 ± 0.5^c^ | 71.7 ± 1.1^b^ | 65.6 ± 1.5^a^ | **16.634 ***** |
|  |  |  |  |  |  |  |  |  |  |  |  |  |  |  |  |  |
| pH at 30 cm |  | 69.7 ± 1.3^a^ | 71.5 ± 1.9^ab^ | 80 ±  0.5^b^ | 4.041 * |  | 68.8 ±  2.6 | 72.0 ± 1.3 | 72.8 ± 2.8 | 67 | 0.745 |  | 79.6 ± 0.5^c^ | 72.6 ± 1.1^b^ | 66.1 ± 1.4^a^ | **17.875 ***** |
|  |  |  |  |  |  |  |  |  |  |  |  |  |  |  |  |  |
| Sand content 15 cm |  | 39.4 ±  1.0 | 41.9 ±  2.1 | 42.3 ± 0.3 | 0.948 |  | 38.5 ±  1.0 | 40.3 ± 1.1 | 43.4 ± 2.9 | 37 | 1.287 |  | 42 ±  1.7 | 41.6 ± 1.6 | 38.3 ± 1.1 | 1.678 |
|  |  |  |  |  |  |  |  |  |  |  |  |  |  |  |  |  |
| Sand content 30 cm |  | 37.8 ±  1.1 | 40.2 ±  2.2 | 40 ±  0.5 | 0.661 |  | 37 ±  1.2 | 38.6 ± 1.2 | 41.7 ± 2.9 | 35 | 1.126 |  | 39.8 ± 2.1 | 39.2 ± 1.6 | 37.8 ± 1.3 | 0.319 |
|  |  |  |  |  |  |  |  |  |  |  |  |  |  |  |  |  |
| Slope |  | 4.8 ±  0.8 | 3.8 ±  0.7 | 4.4 ±  1.1 | 0.251 |  | 4.7 ±  1.3 | 4.4 ±  0.8 | 4.1 ±  0.9 | 7 | 0.228 |  | 3.7 ±  0.8 | 3.4 ±  0.5 | 6.1 ±  1.2 | 2.696 |
|  |  |  |  |  |  |  |  |  |  |  |  |  |  |  |  |  |
| Texture at 15 cm |  | 6.0 ± 0.3^ab^ | 7 ±  0^b^ | 5 ±  1^a^ | 3.731 * |  | 5.8 ±  0.5 | 6.0 ±  0.3 | 7 ± 0 | 7 | 1.208 |  | 5.2 ±  0.7 | 6.6 ±  0.2 | 6.2 ±  0.3 | 2.285 |
|  |  |  |  |  |  |  |  |  |  |  |  |  |  |  |  |  |
| Texture at 30 cm |  | 6.8 ±  1.0^a^ | 5.8 ±  0.4^a^ | 18.6 ± 0.3^b^ | **14.152 ***** |  | 9.5 ±  2.3 | 7.0 ±  1.1 | 7.2 ±  1.8 | 4 | 0.561 |  | 19 ± 0.5^b^ | 5.1 ± 0.3^a^ | 6 ±  0.4^a^ | **187.34 ***** |
|  |  |  |  |  |  |  |  |  |  |  |  |  |  |  |  |  |
| Cation exchange capacity 15 cm |  | 20 ±  0.8 | 17.3 ±  0.8 | 19.6 ± 0.8 | 2.413 |  | 21.2 ±  1.1 | 19.0 ± 0.8 | 16.8 ± 0.9 | 19 | 2.503 |  | 18.4 ± 1.2^a^ | 17.1 ± 0.4^a^ | 21.9 ± 0.9^b^ | **12.144 ***** |
|  |  |  |  |  |  |  |  |  |  |  |  |  |  |  |  |  |
|  |  |  |  |  |  |  |  |  |  |  |  |  |  |  |  |  |
| PC1 |  | -1.021 ± 1.018^a^ | 0.259 ± 0.715^ab^ | 5.603 ± 0.21^b^ | 4.150 * |  | -2.037 ± 1.735 | 0.494 ± 0.985 | 1.308 ± 1.235 | -0.758 | 0.995 |  | 4.869 ± 0.499^c^ | 1.664 ± 0.508^b^ | -4.108 ± 0.841^a^ | **34.439 ***** |
|  |  |  |  |  |  |  |  |  |  |  |  |  |  |  |  |  |
| PC2 |  | -0.483 ± 0.531^a^ | 1.708 ± 0.467^b^ | -2.637 ± 0.327^a^ | 6.769 ** |  | -0.88 ± 0.568 | 0.032 ± 0.663 | 0.94 ± 0.917 | -0.048 | 0.728 |  | -0.906 ± 1.385 | 0.666 ± 0.654 | -0.455 ± 0.475 | 1.215 |
|  |  |  |  |  |  |  |  |  |  |  |  |  |  |  |  |  |
| Niche_suitability _perc2mean |  | 75.526 ± 5.486 | 54 ± 9.713 | 68.333 ± 14.53 | 2.196 |  | 76.25 ± 9.899 | 73.75 ± 5.836 | 43.571 ± 9.619 | 85 | 2.946 |  | 79 ± 10.296 | 64.333 ± 5.973 | 68.333 ± 9.796 | 0.526 |

**Figure S1**. Boxplots of genetic diversity indices averaged across populations, calculated from a jackknife analysis taking increasingly large subsamples of data, from 5 to 12 individuals per population.

**
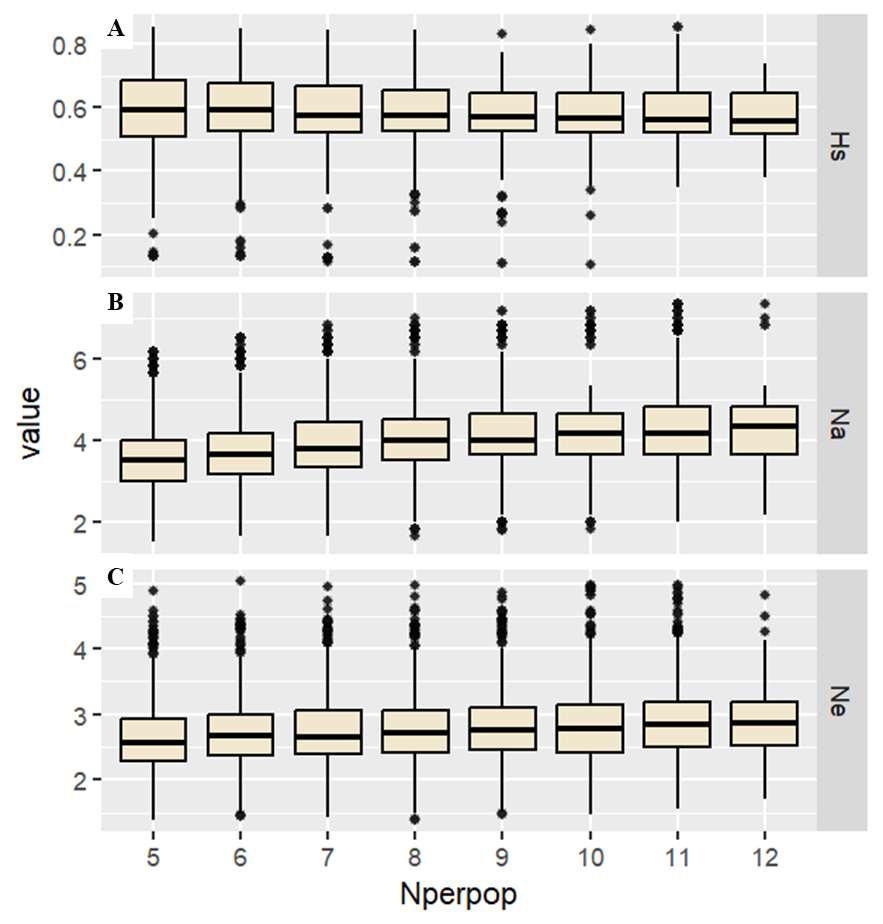
**

**Figure S2**. Delta-K plots estimating the optimal number of genetic clusters following Evanno et al. (2005) for the 32 populations involved in this study (A) and the 29 populations assigned to the Cluster 1 in the former analysis (B).


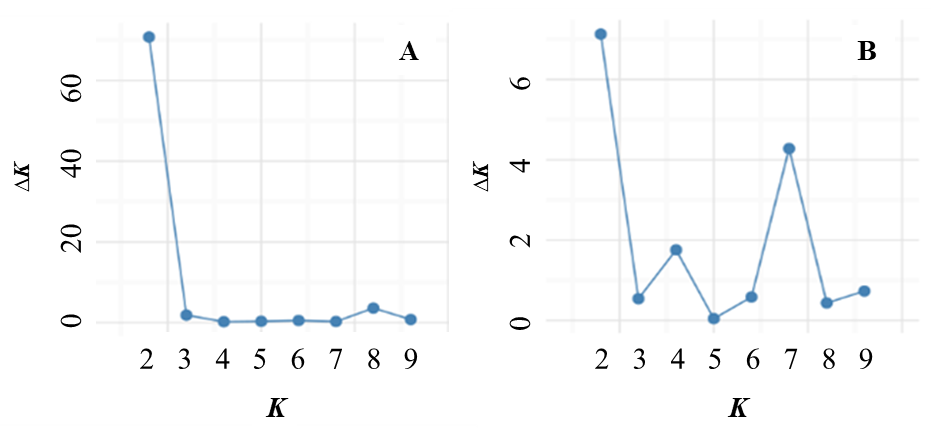

Supplement: plae027_suppl_Supplementary_Tables_S1-S10_Figures_S1-S2_Data_S1 [file plae027_suppl_supplementary_tables_s1-s10_figures_s1-s2_data_s1.zip › 20240328_SSRLsuffruticosum_suppmat.docx]
